# Modelling bluetongue risk in Kazakhstan

**DOI:** 10.1186/s13071-021-04945-6

**Published:** 2021-09-25

**Authors:** Sarsenbay K. Abdrakhmanov, Kanatzhan K. Beisembayev, Akmetzhan A. Sultanov, Yersyn Y. Mukhanbetkaliyev, Ablaikhan S. Kadyrov, Altay Y. Ussenbayev, Aigerim Y. Zhakenova, Paul R. Torgerson

**Affiliations:** 1 Saken Seifullin Kazakh Agrotechnical University, Nur-Sultan (Astana), Kazakhstan; 2 Kazakh Scientific Research Veterinary Institute, Almaty, Kazakhstan; 3 Section of Epidemiology, Vetsuisse Faculty, University of Zürich, Zürich, Switzerland

**Keywords:** Bluetongue, *Culicoides*, Epidemiology, Transmission Kazakhstan, Livestock, Sheep, Cattle, Risk, Basic reproduction number, Geographical information systems, Mathematical modelling

## Abstract

**Background:**

Bluetongue is a serious disease of ruminants caused by the bluetongue virus (BTV). BTV is transmitted by biting midges (*Culicoides* spp.). Serological evidence from livestock and the presence of at least one competent vector species of *Culicoides* suggests that transmission of BTV is possible and may have occurred in Kazakhstan.

**Methods:**

We estimated the risk of transmission using a mathematical model of the reproduction number *R*_0_ for bluetongue. This model depends on livestock density and climatic factors which affect vector density. Data on climate and livestock numbers from the 2466 local communities were used. This, together with previously published model parameters, was used to estimate *R*_0_ for each month of the year. We plotted the results on isopleth maps of Kazakhstan using interpolation to smooth the irregular data. We also mapped the estimated proportion of the population requiring vaccination to prevent outbreaks of bluetongue.

**Results:**

The results suggest that transmission of bluetongue in Kazakhstan is not possible in the winter from October to March. Assuming there are vector-competent species of *Culicoides* endemic in Kazakhstan, then low levels of risk first appear in the south of Kazakhstan in April before spreading north and intensifying, reaching maximum levels in northern Kazakhstan in July. The risk declined in September and had disappeared by October.

**Conclusion:**

These results should aid in surveillance efforts for the detection and control of bluetongue in Kazakhstan by indicating where and when outbreaks of bluetongue are most likely to occur. The results also indicate where vaccination efforts should be focussed to prevent outbreaks of disease.

**Graphical abstract:**

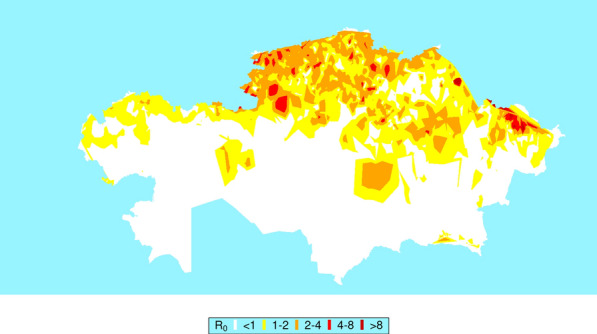

**Supplementary Information:**

The online version contains supplementary material available at 10.1186/s13071-021-04945-6.

## Background

Bluetongue (BT) is a viral disease of ruminants caused by bluetongue virus (BTV), which has now been identified on all continents except Antarctica [1–5]. Hematophagous midges in the genus *Culicoides* are biological vectors that transmit BTV from infected to susceptible ruminants; thus the global distribution of BTV coincides with the distribution of competent *Culicoides* insect vectors and appropriate climatic conditions. The extrinsic incubation period for the virus in the insect vector is many weeks at the minimum temperature for replication of the virus which is 11–13 °C. At higher temperatures, this is reduced to about 5 days at 25 °C and 2.5 days at 35 °C [6]. Specifically, BTV exists in an extensive band that includes tropical, subtropical, and temperate regions of the world between latitudes of approximately 40° north and 35° south [5]. Exceptions include regions of Asia and western North America, where BTV infection of cattle and sheep occurs as far as 50° north [7, 8] and, most recently, northern Europe. Serologically positive animals have been previously reported in Kazakhstan [9] and in Xinjiang in neighbouring China [4]. Cattle imported from Russia into Kazakhstan have also been reported as seropositive [10]. Cattle are usually asymptomatic carriers after being infected with BTV, but can be a potential virus reservoir and source of infection on the farm [4]. The present distribution and risk of bluetongue in Kazakhstan is unknown. The extent of suitable vectors of the virus is also unknown, although the vector-competent species *Culicoides obsoletus* has been found in eastern Kazakhstan [11, 12]. Adult *Culicoides* are killed by cold winter temperatures, and BTV infections typically do not last for more than 63 days [13], which is not long enough for BT-infected animals to remain infectious to the vectors for the duration of the winter. However, in inner Mongolia, which also has cold winters, BT appears to be endemic [14], indicating that the virus can continue to transmit following long periods when the vector in inactive. Therefore, it seems that the virus somehow survives in overwintering midges or animals. A number of mechanisms have been speculated. These include levels of vector activity during mild winters, low numbers of chronic or latent infections in cattle, and horizontal or vertical transmission between cattle. Less likely mechanisms include alternative but unknown reservoir hosts or alternative vectors that are better able to overwinter [15].

The risk of transmission is believed to be affected by the ruminant density, summer temperature, and rainfall (see reproduction number in the “Methods” section). Density of livestock affects the transmission between animals and the *Culicoides* vector, whilst higher temperatures and rainfall are required for vector activity. For example, *C. obsoletus*/*C. scoticus* occurs in regions where annual rainfall is greater than 700 mm, and adult activity starts when the temperature rises above 10 °C [16]. Thus, an important part of developing a risk map for Kazakhstan is to model climate data with livestock density to estimate risk of transmission. Therefore, the objective of this study was to combine this data with a model for the basic reproduction model to indicate the geographical and seasonal risk of transmission in Kazakhstan.

## Methods

### Basic reproductive number of bluetongue

The basic reproduction number, *R*_0_, is defined as the expected number of secondary cases caused by one infectious individual introduced into a naïve population. *R*_0_ is a measure of the success of invasion into a population; if the value of *R*_0_ is higher than 1, an outbreak of the infectious agent is possible, whereas if *R*_0_ is less than 1, the infection is likely to die out [17]. Maps indicating the value of *R*_0_ can be used to identify areas with a higher probability of a major outbreak after an introduction. This concept has been used to develop risk maps for directly transmitted diseases such as foot-and-mouth disease [18]. Therefore, to model the areas of risk for BT in Kazakhstan, established models for *R*_0_ were used.

The basic reproduction number of BT has been defined by [19] as:

1 $$R_{0} = \sqrt {\frac{{a^{2} bcqvh_{c} }}{{\gamma_{c} \left( {h_{c} + h_{s} } \right)^{2} \mu \left( {q + \mu } \right)}} + \frac{{a^{2} bcqvh_{s} }}{{\gamma_{c} \left( {h_{c} + h_{s} } \right)^{2} \mu \left( {q + \mu } \right)}}}$$

**Fig. 1:**
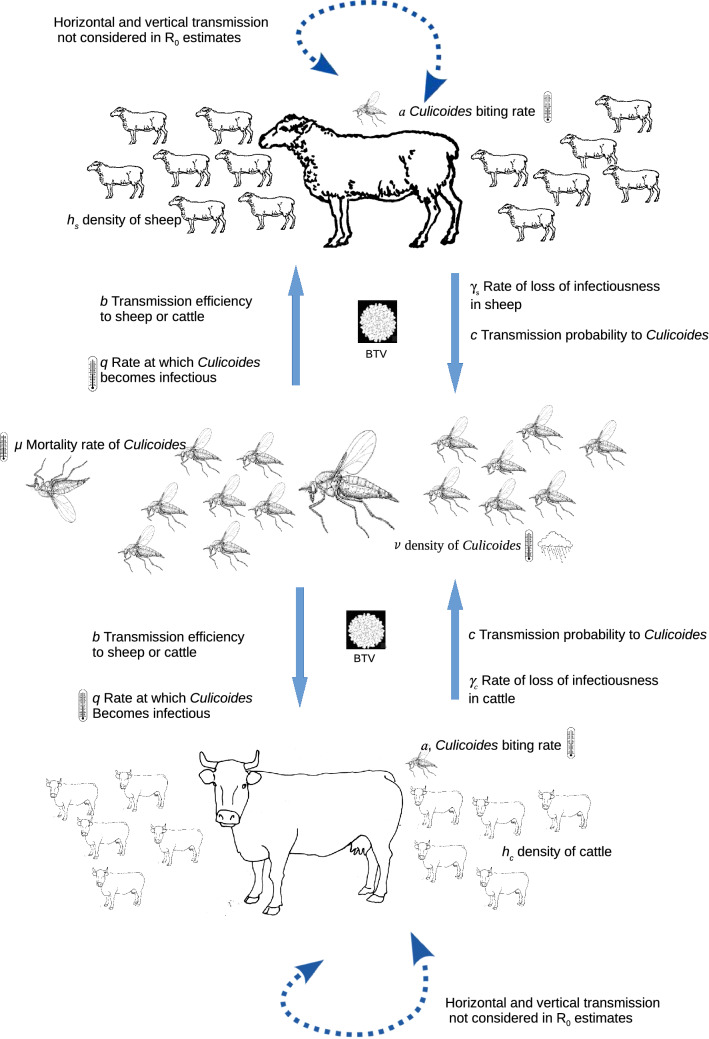
Pictorial representation of the parameters that affect *R*_0_. Parameters affected by temperature and rainfall are indicated

This is where *c* = the transmission probability following a bite by an infected *Culicoides*, *γ*_c_ and *γ*_s_ are the rate of loss of infectiousness in cattle and sheep, respectively (1/*γ* is the duration of infectiousness), *v* the local density of *Culicoides*, *a* is the biting rate (which equals the reciprocal of the length of the gonotrophic cycle), *h*_s_ and *h*_c_ are the population densities of sheep and cattle, respectively, *c* is the transmission probability from cattle or sheep to *Culicoides* following a bite, *b* is the transmission efficiency from *Culicoides* to host, *q* is the rate at which the *Culicoides* becomes infectious, and *μ* is the *Culicoides* mortality rate. Furthermore, *a*, *q*, and *μ* are temperature-dependent, and *v* is dependent on climatic conditions. The values for the various parameters are given in Table 1. A pictorial representation is illustrated in Fig. 1.

**Table 1:** Parameter values for the basic reproductive ratio

Parameter	Value
*b*	1
c	0.05
γ_c_	0.04
γ_s_	0.125
*v*/100	log_e_(*v* + 1) ~ −0.856 + 0.076T_i,i_ + 0.048T_i-37,i_ + 2.913P_i-100j-16_
*a*	*a* ~ 0.00017 T(T − 3.7)(41.9 − T)^1/2.7^
*q*	*q* ~ 0.0003T_max_(T_max_−10.4)
*μ*	*μ* ~ 0.09exp(0.16 T)

T = average mean temperature of the month in question

The relationship between vector density *v* and temperature and precipitation was from [20], where *T*_i_ is the daily temperature (in this case, the mean monthly temperature), and *T*_i-37i_ is the mean temperature for the proceeding 37 days (in this case the mean temperature of the preceding month)

*T*_max_ is the maximum daily temperature, and *P*_i-100j-16_ the mean precipitation for the preceding days 100 to 16. All other parameters are from [19]

### Data

Kazakhstan is divided into 14 first administrative level regions (or oblasts), and these are subdivided into 170 second administrative level districts (or rayons). The rayons are further subdivided into 2466 third administrative level village districts (or selski okrugs, SO). Data for the cattle and small ruminant populations were provided to the third administrative level [21]. This data which gives total populations of cattle and small ruminants for each village district is provided in Additional file 1: Dataset S1. Small ruminant populations were not disaggregated into sheep and goat populations, so the total small ruminant population reported was used to estimate the sheep population density in Eq. 1; this resulted in 2466 data points. The geographical area of each SO was also obtained from [21], and this together with the sheep and cattle populations reported it was possible to estimate the livestock density for each SO (i.e. *h*_c_ and *h*_s_ in Eq. 1). Climatic data was available for the main settlement at the second administrative level, and this was used for each of the village settlements in that rayon. This climatic data was obtained from [22] and is based on 30-year averages between 1982 and 2012. This climatic data, together with the livestock data, coordinates, and area of each of the third administrative district is also given in the additional data file.

### Analysis and mapping

All data was entered into an Excel spreadsheet; subsequently, analysis and mapping was completed using R statistical software [23]. The basic reproductive number was estimated for each SO from Eq. 1. This was based on the climatic data and livestock populations. *R*_0_ for each SO was calculated for each month of the year. The resultant data grid of 2466 data points was smoothed by linear interpolation [24], and isopleth maps were drawn. When *R*_0_ is below 1, disease transmission cannot be maintained. When *R*_0_ > 1, the proportion of the population immune to the disease (level of herd immunity) to prevent an outbreak of disease is 1-1/*R*_0_ [25]. Therefore, this also defines the proportion of the population required to be vaccinated to prevent disease outbreaks. For each of the 2466 data points, we estimated the maximum *R*_0_ from April to October and used this to estimate the proportion of the livestock population required to be vaccinated to prevent outbreaks of BT. These were also plotted on an isopleth map. The maps were drawn in R using ggplot2 [26]. R code and associated files are provided in Additional file 2: Dataset S2. We also undertook sensitivity analysis to see how varying the parameters *b*, *c*, *γ*_c_, and γ_s_ affected the number of village communities that had *R*_0_ > 1 during July which was the peak month for potential transmission (see Table 2).

**Table 2:** Sensitivity analysis

b	*N*	c	*N*	*g*_c_	*N*	*g*_s_	*N*
1*	1422*	0.05*	1422*	0.04*	1422*	0.125*	1422*
0.5	1103	0.025	1103	0.02	1578	0.0625	1552
0.3	829	0.0125	735	0.012	1691	0.0375	1669
0.2	615	0.01	625	0.008	1772	0.025	1757
0.1	265	0.005	265	0.004	1887	0.0125	1895

Number of villages (*N*) in July with *R*_0_ > 1) by varying the parameters b, c, γ_c_, γ_s_

*Baseline scenario used in the model

One parameter was varied in each scenario, and the other parameters held at baseline

## Results

**Fig. 2:**
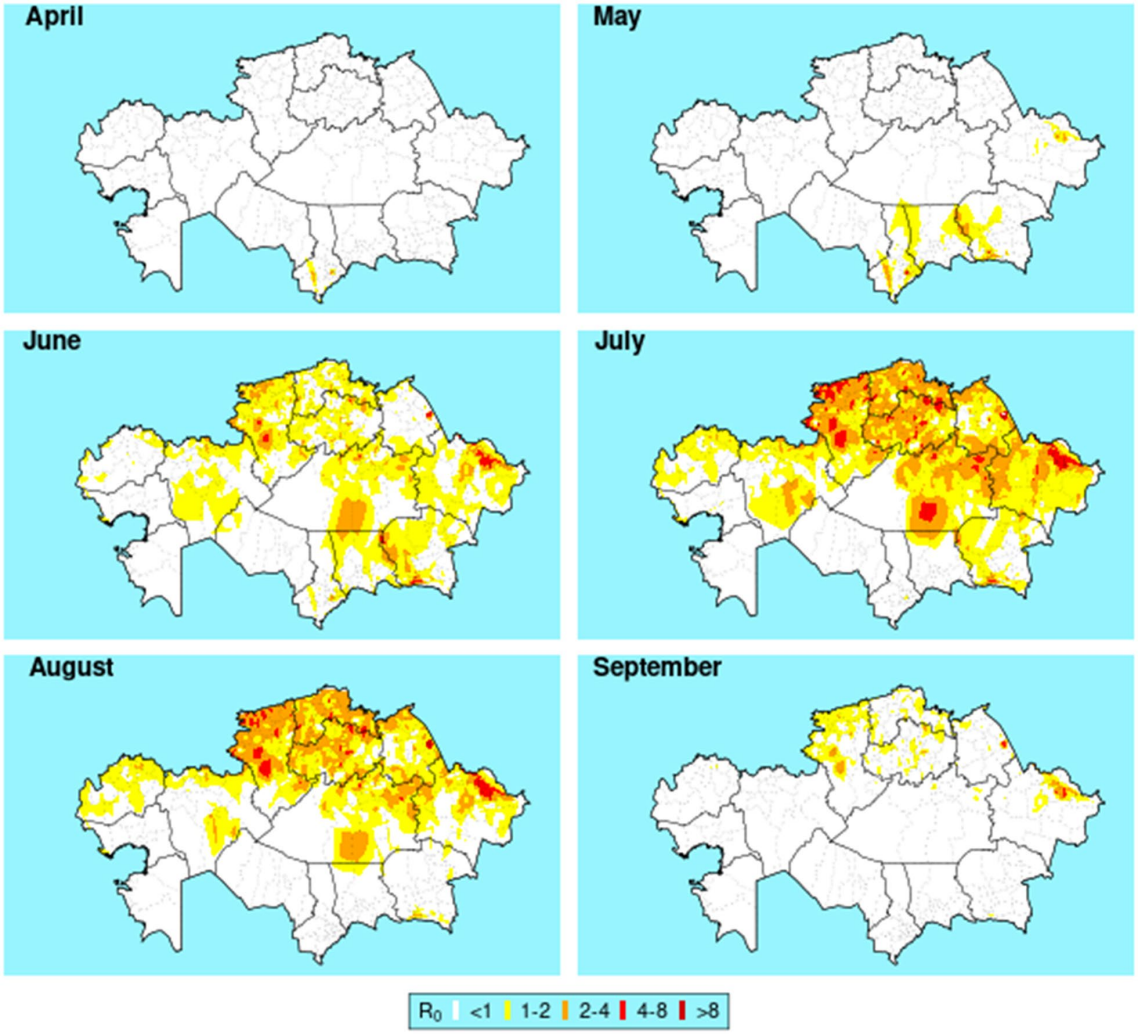
*R*_0_ of transmission of bluetongue in Kazakhstan from April to September

The *R*_0_ for BT transmission is displayed for the months April–September. The model indicated that outbreaks were unlikely to occur from October to March. Risk of outbreaks appeared initially mainly in the south Kazakhstan region. This spread across the southern regions, South Kazakhstan, Jambyl, and Almaty, in May. By June, outbreaks were possible in the northern parts of Kazakhstan. *R*_0_ and hence potential outbreaks peaked in July in the north and north-east regions of Kazakhstan, by which time *R*_0_ was starting to decrease in the south. In September, *R*_0_ was decreasing in all regions and in many areas had been reduced below the threshold of transmission. By October, *R*_0_ was below 1 in all areas of Kazakhstan. Estimated *R*_0_ transmission for the months of April to September is illustrated in Fig. 2.

**Fig. 3:**
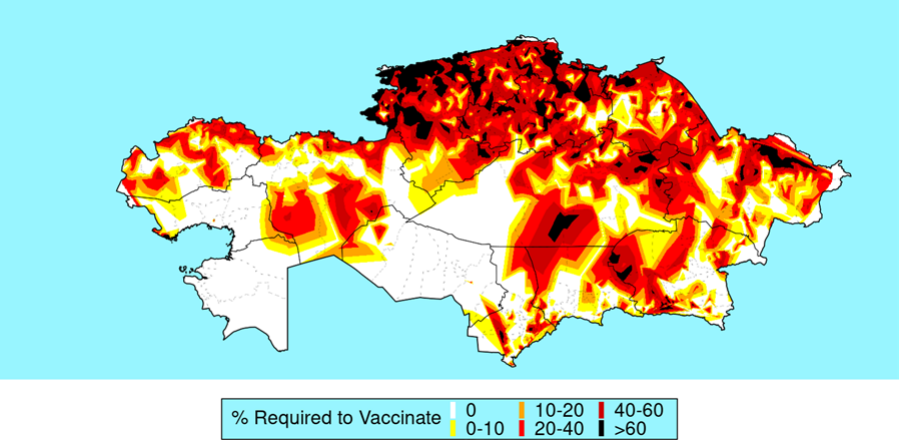
Estimated proportion of livestock requiring vaccination to prevent outbreaks of bluetongue

The estimated proportion of livestock that need to be vaccinated is illustrated in Fig. 3. There are large parts of the country in the centre and south-east of the country where vaccination is not required, as *R*_0_ is below 1 throughout the year. In contrast, in the north, vaccination of most animals would be required if the virus was introduced.

Sensitivity analysis indicated that the area in which *R*_0_ remained above 1 was fairly robust to changes in b, c, γ_c_, and γ_s_. At the baseline scenario with the parameters used as defined in Table 1, there were 1422 districts (of 2466) where *R*_0_ was > 1 in July. Even halving individual parameters only resulted in the number of districts with *R*_0_ > 1 varying by approximately 10–23% (Table 2).

## Discussion

The results obtained clearly indicate there is a heterogeneity of risk, both geographically and temporally, of BT outbreaks in Kazakhstan. This assumes that the BTV can only be transmitted by a vector. There is some evidence for possible transmission by direct contact between animals [27, 28], and it is not known whether this transmission pathway can contribute to outbreaks of disease. Across the whole country, in winter months the risk is negligible. This is because it is too cold for the vector to be active, as mean temperatures across most of Kazakhstan are below freezing from November to March. In April, temperatures are high enough for vector activity in the south, and this spreads north, with peak transmission in northern areas in July. In addition, the activity of vectors is dependent on rainfall [16]. Southern areas such as around Shymkent have relatively low rainfall in the summer months from June to September compared to the wetter spring months of March to May, which will be a factor in the reduced transmission in these areas. In contrast, Petropavl in the north has its highest rainfall in the summer months. Much of central Kazakhstan has very low rainfall year-round, which is associated with the low risk of transmission in these areas, with *R*_0_ remaining below 1 in many areas. Vaccines are available to prevent BT [29], and our results suggest that to prevent outbreaks of disease, livestock vaccination should be targeted in the northern regions of Kazakhstan. Sensitivity analysis indicated that *R*_0_ is stable across a range of transmission probabilities and efficiencies and the duration of infectiousness of cattle or sheep, and thus uncertainty in these parameters is unlikely to have a large influence on the conclusions. Other parameters were fixed that is temperature rainfall and livestock density.

This analysis was based on the *R*_0_ associated with the BTV vector *Culicoides*. The model for *R*_0_ was a generic model which was not species-specific [19], but the temperature and rainfall dependency of the vector population was based on work for *C. obsoletus.* A potential weakness of the study is that whilst we used the logistic regression model of [20] to model *v*, the local density of *Culicoides,* we had to use mean monthly temperatures for the current month and previous month and mean precipitation data as proxies for the actual daily temperature and mean temperatures and precipitation. This was due to availability of the data, and we attempted to project possible risk of outbreaks based on long-term climatic factors rather than the risk described over a small number of seasons. Despite this limitation*,* specimens of *C. obsoletus* have been reported in various regions of Kazakhstan [11, 12], and hence a model utilizing methods based on *C. obsoletus* would be the most relevant to this study.

There are a number of other *Culicoides* spp. endemic to the region. A review by Sprygin et al. [30] suggested that 23 species of *Culicoides* have been detected in Kazakhstan, including widespread species such as *C. reconditus*, *C. fascipennis*, and *C. vexans*. *C. brevifrontis* and *C. manchuriensis* have been found in Akmola Oblast, in north-central Kazakhstan [31]. Although there are well over 1000 species of *Culicoides*, only about 30 are believed to be vector-competent for BTV [32]. The duration of viraemia is less than 2 months in small ruminants and cattle [33, 34], and this presents a possible limiting factor for transmission, as the virus should not be able to overwinter in cattle or sheep when there is no *Culicoides* activity. However, it has been shown that BTV virus can persistently infect ovine γδ T-cells, and this might provide a mechanism for virus persistence and overwintering [35]. BTV nucleic acid has been detected in field-collected *Culicoides* spp. larval pools. This supports the hypothesis that the virus can overwinter in vertically infected immature life stages of the vector [36]. In Southern Europe, Turkey, and Iran, *C. imicola* is an important vector of BTV [37], and in the warmer parts of these regions, the short viraemia in farm animals does not limit the transmission of BTV because of the more favourable climatic conditions. Specimens of *C. imicola* have not been reported in Kazakhstan, although it has been found in northern Iran [38] just across the Caspian Sea. Modelling has suggested that favourable conditions for *C. imicola* may be present in the southern most parts of Kazakhstan [39]*.* Other vectors may be implicated in the spread of BTV northwards in Europe [40]. For example, *C. scoticus*, in addition to *C. obsoletus*, has been shown to be an efficient vector of BTV [41].

Transmission in Switzerland at altitudes as high as 1500 m in Lenzerheide in Canton Grisons appears to be possible [41]. There, winter minimums are −10 °C, summer maximums are 18 °C, and there are only 2 months with a mean temperature above 12.5 °C. In Lenzerheide, the conditions compare unfavourably to most of southern Kazakhstan, and summers are cooler and shorter than in northern Kazakhstan. For example, in Shymkent, in the far south of Kazakhstan, there are 7 months with a mean temperature above 12.5 °C, and winter minimums average −6 °C, whilst summer highs average 34 °C. In Petropavl, which borders southern Siberia and is 2000 km farther north, winter lows average −21 °C with summer highs averaging 25 °C with just 3 months where the mean temperature is above 12.5 °C. In addition, there is some evidence that the virus may be able to overwinter in northern Europe [15]. This suggests that if vector-competent species of *Culicoides* are endemic to Kazakhstan, then there is a risk of outbreaks of BT, and the data displayed in Fig. 2 would represent the risk of outbreaks. However, in regions where there is the absence of vector-competent species, then outbreaks of BT should not be possible. Vector-free vertical and horizontal transmission has been reported, but may only be relevant to local on-farm transmission [42]. It is worth noting that serological evidence of BTV has been previously reported in Kazakhstan with a seropositivity of 25% of 279 cattle and 22% of small ruminants (sheep and goats) from central and southern Kazakhstan in the period 1996–1998 [9]. Thus, if true, there are likely to be vector-competent species in Kazakhstan and a mechanism other than viraemia in livestock by which the virus can persist through the winter and transmit from one season to the next. *C. obsoletus* has been reported in some districts of Kazakhstan [11, 12], such as East Kazakhstan. Seropositive cattle which had been imported from Russia were found in this region of Kazakhstan [13], illustrating the risk of outbreaks. However, the extent and distribution of this and other vector-competent species is unknown and, therefore, represents an important data gap for understanding the risk of transmission of BTV in Kazakhstan.

## Conclusions

Presently, there have been no recent outbreaks of BT in Kazakhstan. This study shows the high-risk districts for outbreaks of BT should the disease appear and thus helps with epidemic preparedness. In June 2006, BTV appeared in northern Europe for the first time, successfully overwintered, and subsequently caused substantial losses to the farming sector in 2007 and 2008 [43]. This illustrates the need for such preparedness.

## Supplementary Information

**Additional file 1:Dataset S1.** Data on small ruminant and cattle population for each third level village district, together with the area and coordinates of the district. Climatic data for mean monthly temperature and rainfall is based on the data from the principal settlement in the rayon.

**Additional file 2:Dataset S2.** R code and associated files.

## Data Availability

All data used in the analysis can be obtained elsewhere [21, 22]. These data are also provided as Additional file 1: Dataset S1 and Additional file 2: Dataset S2.

## Abbreviations

BT: Bluetongue
BTV: Bluetongue virus
SO: Selski okrugs
R_0_: Reproduction number
