# Supplementary material for: Modelling bluetongue risk in Kazakhstan
Source: Parasit Vectors. 2021 Sep 25;14:491. doi: 10.1186/s13071-021-04945-6 (PMC8465711; doi:10.1186/s13071-021-04945-6)
Supplement: Supplementary file 2 — Additional file 2:Dataset S2. R code and associated files. [file 13071_2021_4945_MOESM2_ESM.zip › data files/readme.docx]

The files need to be placed in a single folder to run the code.

The files include the shape files for Kazakhstan including administrative level 1 and 2 boundaries.

Estimated R0 for the months April-September are given in the .csv files, together with the coordinates of community where each R0 values was estimated.

year1.csv also contains the R0 for each month and the maximum R0 estimated for each community from April to September. This is used to generate figure 2.
